# Creosote growth rate and reproduction increase in postfire environments

**DOI:** 10.1002/ece3.5771

**Published:** 2019-10-25

**Authors:** Rebecca Lee Molinari, Tara B. B. Bishop, Matthew F. Bekker, Stanley G. Kitchen, Loreen Allphin, Samuel B. St. Clair

**Affiliations:** ^1^ Department of Plant and Wildlife Sciences Brigham Young University Provo Utah; ^2^ Department of Geography Brigham Young University Provo Utah; ^3^ United States Department of Agriculture, Forest Service Rocky Mountain Research Station Provo Utah

**Keywords:** creosote bush, dendrochronology, fire ecology, *Larrea tridentata*, Mojave Desert

## Abstract

Human activities are changing patterns of ecological disturbance globally. In North American deserts, wildfire is increasing in size and frequency due to fuel characteristics of invasive annual grasses. Fire reduces the abundance and cover of native vegetation in desert ecosystems. In this study, we sought to characterize stem growth and reproductive output of a dominant native shrub in the Mojave Desert, creosote bush (*Larrea tridentata* (DC.) Coville) following wildfires that occurred in 2005. We sampled 55 shrubs along burned and unburned transects 12 years after the fires (2017) and quantified age, stem diameter, stem number, radial and vertical growth rates, and fruit production for each shrub. The shrubs on the burn transects were most likely postfire resprouts based on stem age while stems from unburn transects dated from before the fire. Stem and vertical growth rates for shrubs on burned transects were 2.6 and 1.7 times higher than that observed for shrubs on unburned transects. Fruit production of shrubs along burned transects was 4.7‐fold more than shrubs along paired unburned transects. Growth rates and fruit production of shrubs in burned areas did not differ with increasing distance from the burn perimeter. Positive growth and reproduction responses of creosote following wildfires could be critical for soil stabilization and re‐establishment of native plant communities in this desert system. Additional research is needed to assess if repeat fires that are characteristic of invasive grass‐fire cycles may limit these benefits.

## Graphical abstract

Our data support the conclusion that postfire environments increase creosote growth rates (Figure 3) and increase fruit number (Figure 4). However, distance to the fire perimeter did not affect growth rates or fruit number.

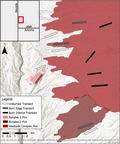

## 1 INTRODUCTION

Wildfires strongly influence plant community composition, biodiversity, and function across the Earth's ecosystems (Moritz et al., 2014). Human activities are changing fire regimes globally (Bowman et al., 2011) through land‐use change, fire suppression, fire ignition, and climate change (Flannigan, Krawchuk, Groot, Wotton, & Gowman, 2009). North American deserts, which historically experienced fire return intervals on the century time scale, are now experiencing larger fires on shorter time intervals due to the introduction and spread of invasive annual grasses (Brooks et al., 2004). A critical question in the field of ecology is how human‐altered fire regimes are changing the composition and function of native plant communities.

Native shrubs in desert ecosystems tend to be poorly adapted to fire (Abella, 2009; Brown & Minnich, 1986; Horn, Wilkinson, White, & St. Clair, 2015) and take long fire‐free periods to recover. Some shrubs are able to survive and resprout after fire (Abella, Engel, Lund, & Spencer, 2009), though less is known about the growth and reproductive responses of these resprouting shrubs. Studies have examined the effects of reduced competition on growth rates of desert shrubs through mechanically thinning neighboring shrubs and annuals or the effects of fire on regenerating shrubs in other ecosystem types (Holzapfel & Mahall, 1999; Lamont, Enright, & He, 2011; Mahall, Fonteyn, Callaway, & Schlesinger, 2018; McCarron & Knapp, 2003; Radosevich & Conard, 1980). However, the growth rates of resprouting native desert shrubs in burned areas compared to shrubs in unburned areas are not well characterized. Concerning reproductive responses of native desert shrubs to fires, Lybbert, Taylor, Defranco, and St. Clair (2017) found that flower and fruit production in regenerating generalist pollinated species tended to increase in burned areas.

Native plant response varies with burn severity and can be influenced by fuels, topoedaphic context, and weather (Whitman et al., 2018). Depending on burn severity, fire in deserts can lead to a short‐term increase in soil nutrients but can also lead to lower soil moisture and higher soil temperatures due to hydrophobicity and loss of vegetation and litter cover (Allen, Steers, & Dickens, 2011; Esque, Young, & Tracy, 2010; Snyman, 2003). However, more water may become available for regenerating plants after fire due to reduced competition (Brisson & Reynolds, 1994; Horn et al., 2015). Differences in burn severity at fire perimeters may result in edge effects that affect shrub growth responses. For example, shrub density on burn edges was found to be higher than shrub densities in the burn interior after fire in the Mojave Desert (Lybbert et al., 2017). The density of colonizing and resprouting shrubs in postfire arid environments has been shown to be influenced by topographic position of the burn edge, distance from the burn edge, and proximity to seed sources (Condon & Weisberg, 2016). This led us to question if shrub growth and reproduction vary spatially from the edges to the interior of burned landscapes.

The Mojave Desert is located in the southwestern United States and is the smallest desert in North America. The Mojave has experienced an increase in the number and size of fires in recent decades (Brooks & Matchett, 2006) causing changes in plant community structure and soil resource availability (Horn et al., 2015). These adjacent burned and unburned areas provide an opportunity to study native plant regeneration and resource competition. *Larrea tridentata* (DC.) Coville, or creosote bush (hereafter just creosote), is a multistemmed, evergreen species that is well adapted to desert environments and consequently is one of the dominant shrubs in the Mojave and other North American desert shrublands. Creosote can establish through both sexual and asexual reproduction (Chew & Chew, 1965; McAuliffe, Hamerlynck, & Eppes, 2007). This clonal shrub is long‐lived although individual shoots are replaced as aging or drought occurs (Vasek, 1980). Fire results in high mortality rates but if the root system or crown survives, resprouting is known to occur (Abella, 2009). Creosote provides habitat and food for desert fauna and can increase soil nutrient and water supply through fertile islands, thus playing a key role in the ecosystem (Bainbridge & Virginia, 1990). Understanding the growth and reproduction patterns of this shrub in burned and unburned areas is critical to understanding postfire desert ecosystem assembly and function.

The objectives of this study were to examine and assess differences in growth and sexual reproductive effort (fecundity) of regenerating creosote in burned and unburned landscapes. We asked the following questions: (a) How does growth rate for regenerating creosote stems in burned areas differ from that of stems from unburned areas? (b) How does sexual reproductive response (fecundity) differ for postfire regenerating and unburned creosote and how does that difference change over time?, and (c) Does proximity to the fire perimeter (edges vs. interiors of large fires) affect growth rates of burned/regenerating creosote?

## 2 METHODS

### 2.1 Study location

**Figure 1:**
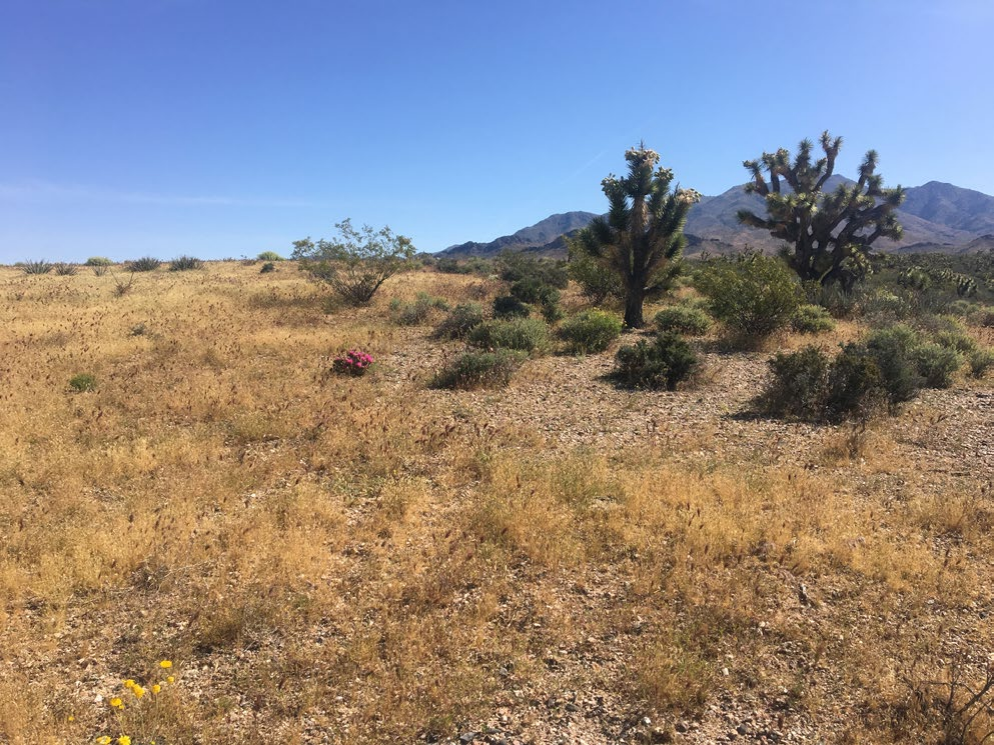
The burned and unburned side of a fire boundary in the northeastern Mojave Desert 14‐year postfire

This study was conducted in the Beaver Dam Wash in the northeastern Mojave Desert (Latitude 37.0837 N, Longitude 114.0119 W, and elevation 1,216 m). The 30‐year mean annual precipitation from the nearest Lytle Ranch Climate Station is 26.5 cm (Western Regional Climate Center, [Link]). Dominant vegetation includes *L. tridentata*, *Yucca brevifolia* Engelm., *Ambrosia dumosa* (A. Gray) Payne, and *Coleogyne ramosissima* Torr.. The landscape has low‐sloping ridges with young alluvial soil with a sandy loam texture. The study area experienced three separate lightning‐caused wildfires in the summer of 2005: Westside Complex (June, 23,782 ha), Burgess 1 (July, 60 ha), and Burgess 2 (July, 543 ha). The fire boundaries were identified using the Monitoring Trends in Burn Severity project (MTBS) and corroborated in the field (Horn et al., 2015; Monitoring Trends in Burn Severity Program, 2017) (Figure 1). Transect analysis at our study area found that creosote density was reduced more than fourfold in burned areas compared to unburned locations (0.8 shrubs per 100 m^−2^ vs. 3.5 shrubs per 100 m^‐2^), but creosote densities did not differ between burned edge or burned interior locations (Lybbert et al., 2017).

### 2.2 Study design

**Figure 2:**
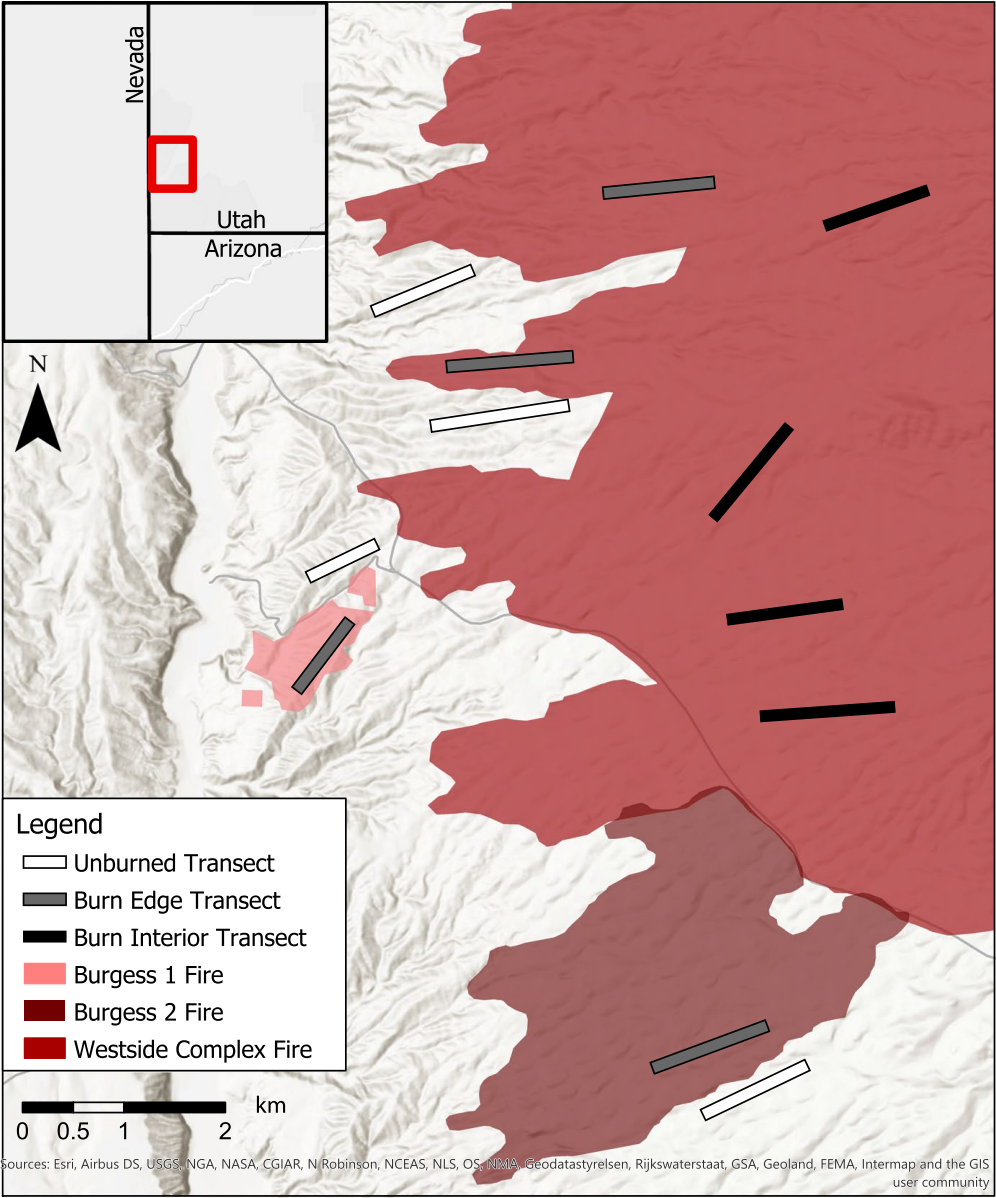
Map of the fire extents and transect locations in the northeastern Mojave Desert study site near the Beaver Dam Wash (Latitude 37.0837 N, Longitude 114.0119 W, and elevation 1,216 m)

Creosote stem growth and sexual fecundity were characterized along four pairs of transects, each 1 km in length, and positioned on the burned and unburned side of fire boundaries of each of the three wildfires (Figure 2). Transects were located within 200 m of the fire boundary to ensure similar physiographic conditions. Four additional transects were located in the interior of the largest fire (Westside complex, >1.5 km from burn perimeter). Paired transects were located along the tops of ridges to help standardize topographic conditions between transects and fires. Burn interiors had less topographical variation. We sampled a single creosote shrub nearest to each 200‐m interval point along each transect line. Study shrubs were tagged for measurement of annual fruit number and plant growth measurements (described below). There were three to five shrubs per transect with 17 total shrubs on the unburned transects, 19 on the burn edge transects, and 19 on the burn interior transects.

### 2.3 Growth measurements and dendrochronology

To assess age and annual radial growth, one stem sample was collected from each of the 55 study shrubs 12 years after the burn (2017). To standardize collection, the longest stem from each shrub was selected. The stem was cut as close to the base of the stem as possible. In the laboratory, stems were trimmed to 5‐cm cross sections, keeping the segment proximal to the root collar. Samples were then surfaced with increasingly finer sandpaper (150 grit‐9 micron) until individual cells could be distinguished using a stereomicroscope to facilitate the determination of ring boundaries. These samples were used for ring count analysis.

Creosote is a diffuse‐porous species, which makes annual growth rings difficult to identify. To account for this, three analysts independently aged each cross section. The ring for the collection year (2017) was counted as a full year. An age estimate was assigned for each stem by averaging the three independent estimates. Observer age estimates differed by four or fewer years for all of the samples from burned areas with over 80% of the samples differing by two or fewer years. Creosote has been observed to resprout in the same year as the fire (Dalton, 1961), hence cross sections with 13 rings were classified as postfire. Stem age estimates from unburned areas had greater count disparities because the stems were older and outer rings were much narrower and less distinct. Therefore, ages for the cross sections from unburned areas may have been underestimated. However, if this is the case, difference in growth rates between the burned and unburned areas would be even more pronounced.

We calculated stem radial growth rate by dividing the average stem diameter by the assigned stem age for each cross section (Kitchen, Meyer, & Carlson, 2015). Average diameter was calculated by averaging the longest diameter and the diameter perpendicular to it dissecting at pith. Stem number was counted in 2019 to further investigate growth rate and was the number of shoots of a shrub that connected at or belowground. Vertical growth rates were calculated by dividing the height of each shrub (measured from the ground to the tallest point) by the age of the sampled stem. All growth measurements for each shrub were averaged across individual transects.

### 2.4 Fruit counts

Fruit production was counted on each study shrub every June from 2015 to 2017. Where fruit numbers were high, the shrub was quartered with 1‐m PVC pipes connected at right angles by a four‐way cross connector. The fruits from a randomly selected quarter were counted and then multiplied by four (Lybbert et al., 2017). The fruit number for each shrub was averaged by transect.

To verify that observed trends in fruit number were not due to differences in shrub size, we calculated fruit density for the 2017 data. Volume was calculated for each shrub using the shape of an inverted cone (Chew & Chew, 1965).

$$\text{volume}=\frac{1}{3}\left(\pi \times \text{major}\;\text{radius}\;\times \;\text{minor}\;\text{radius}\;\times \;\text{height}\right).$$

We divided the fruit number for each shrub by its volume and then averaged those values by transect.

### 2.5 Statistical analysis

Linear mixed effects models were used to test the effects of burn condition (unburned vs. burn edge) as well as burn location (burned edge vs. burned interior) on stem age, stem diameter, stem number, stem radial growth rates, height, vertical growth rates, fruit number, and fruit density. Since fruit counts were collected for more than one year, year and the interaction were also included as fixed effects for the fruit number mixed effects models. Transect pair was used as a random effect in all models. We used data exploration techniques to examine whether model assumptions for normality and equal variance of the residuals were met (Zuur, Ieno, & Elphick, 2010). When the assumptions were not met, the data were square root transformed. All data exploration and statistical analysis were performed in the program R (R Core Team, 2018) with additional car and nlme packages (Fox et al., 2012; Pinheiro, Bates, Debroy, & Sarkar, 2017).

## 3 RESULTS

### 3.1 Shrub age

Creosote along burned transects were on average four years younger than those from unburned transects (Table 1). On average, shrub stems along unburn transects began growing in 2001 (prefire), while burn edge and burn interior shrub stems were dated to 2005 and 2006 just after the fires.

**Table 1:** The average creosote stem age for each transect type from the study site is shown with ± *SE*

Fire	Average stem age (years)
Unburned (U)	16.6 ± 0.5
Burn edge (E)	12.1 ± 0.6
Burn interior (I)	11.1 ± 0.3
U × E	32.1**
E × I	2.9

Note

The lower portion of the table shows the *F* values from the mixed models.

Abbreviations: E, burn edge; I, burn interior; U, unburned.

Significance is denoted with asterisks: ** *p *< .01

### 3.2 Growth rate and fruit number of adjacent burn edge and unburned transects

**Figure 3:**
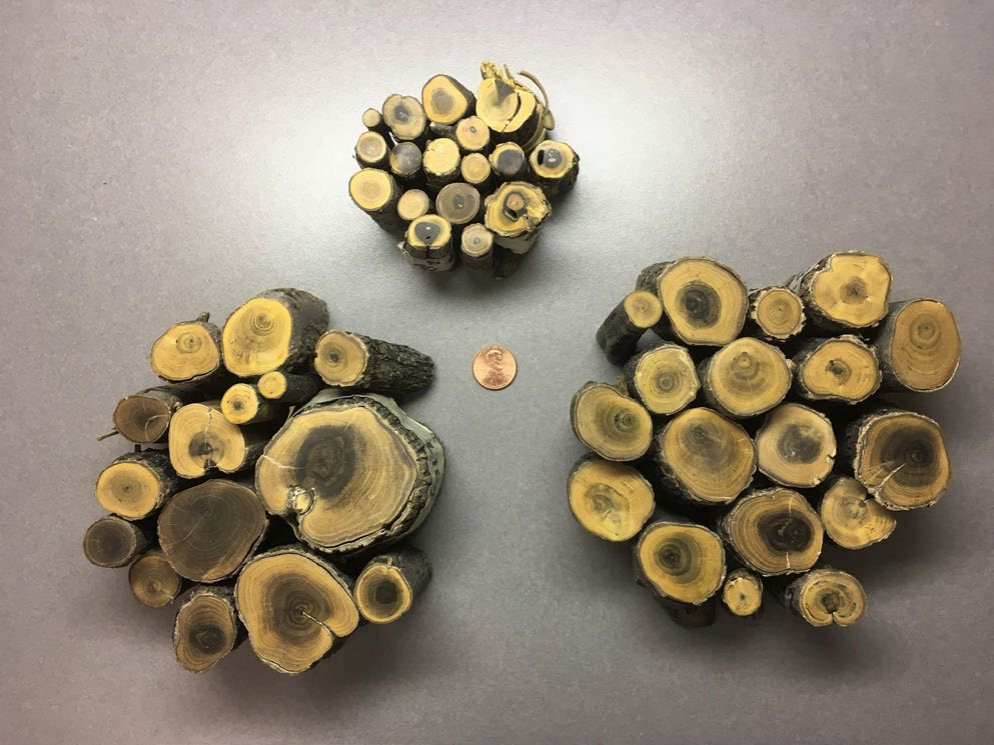
Cross sections from creosote stem samples collected from unburned (top), burn edge (bottom left), and burn interior transects (bottom right)

**Figure 4:**
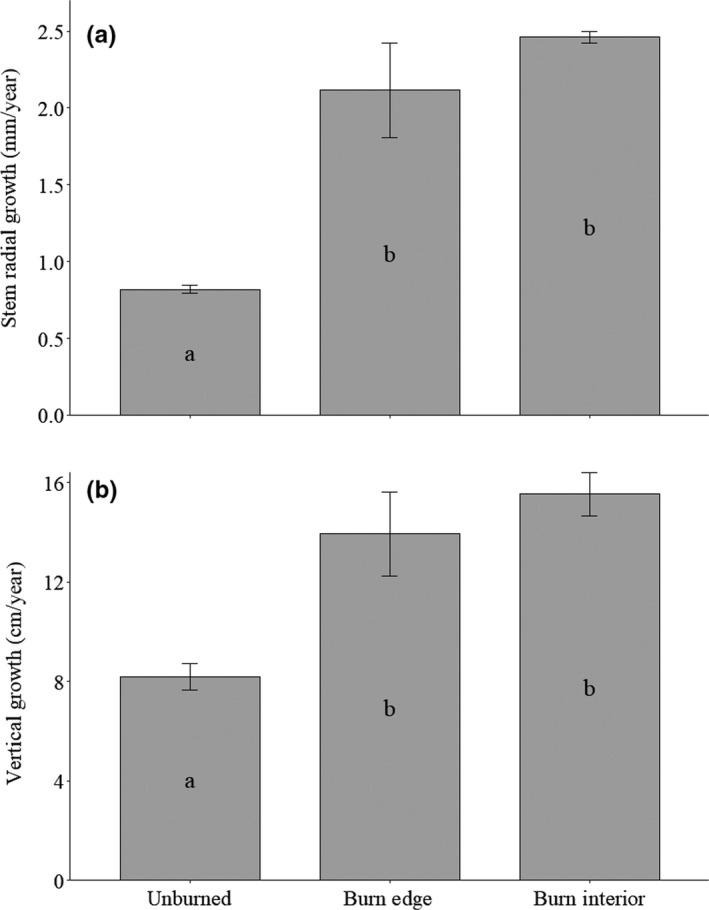
(a) Mean stem radial growth and (b) vertical growth rates ± *SE* of creosote by burn condition and burn location. Significant differences (*p* < .05) are denoted by different letters

Creosote generally had positive growth responses along burned transects compared to adjacent unburned transects (Figure 3). Average sampled stem diameter in burned areas was 25.7 mm, while average sampled stem diameter from unburned areas was 13.6 mm (*F* = 12.0, *p* = .04). The average number of stems from shrubs in burned areas was 9 while those in unburned areas was 20 (*F* = 128.1, *p* = .002). Average creosote stem radial growth rates along the burned edges were 2.6 times greater than in unburned transects (2.1 mm/year vs. 0.8 mm/year, *F* = 31.7, *p* = .01) (Figure 4a). Average vertical growth rates followed a similar pattern with shrubs in burned transects growing 1.7 times more per year than shrubs in unburned transects (13.9 cm/year vs. 8.2 cm/year, *F* = 18.2, *p* = .02) (Figure 4b). The mean heights of shrubs were greater on burned edges compared to unburned edges, though not statistically significant at *p* ≤ .05 (165.3 cm vs. 133.8 cm, *F* = 6.1, *p* = .1).

**Figure 5:**
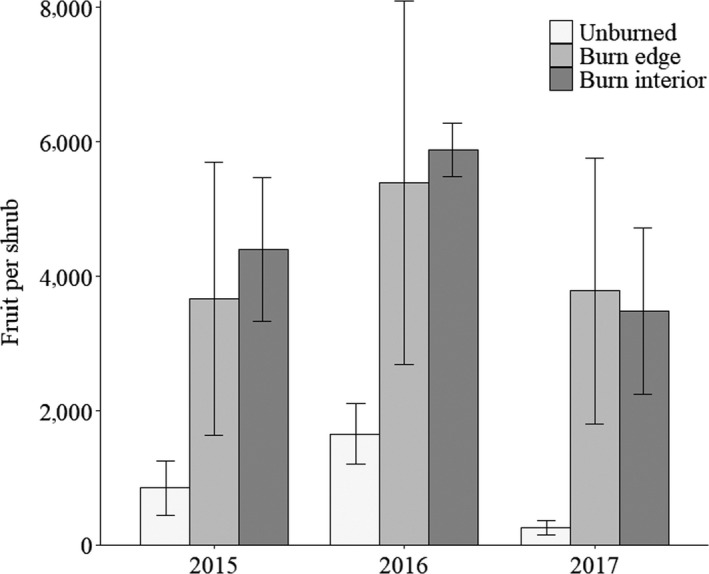
Average fruit number per creosote by burn condition and location for each observed year ± *SE*. Unburned transects compared to burn edge transects (burn condition) had *F* = 18 and *p* = .0007, Year had an *F* = 2.0 and *p* = .2, and Burn condition*Year effect *F* = 0.3 and *p* = .7. Burn edge compared to burn interior (burn location) had *F* = 0.1 and *p* = .7, Year had *F* = 2.1 and *p* = .2, and Burn location*Year had *F* = 0.09 and *p* = .9

**Figure 6:**
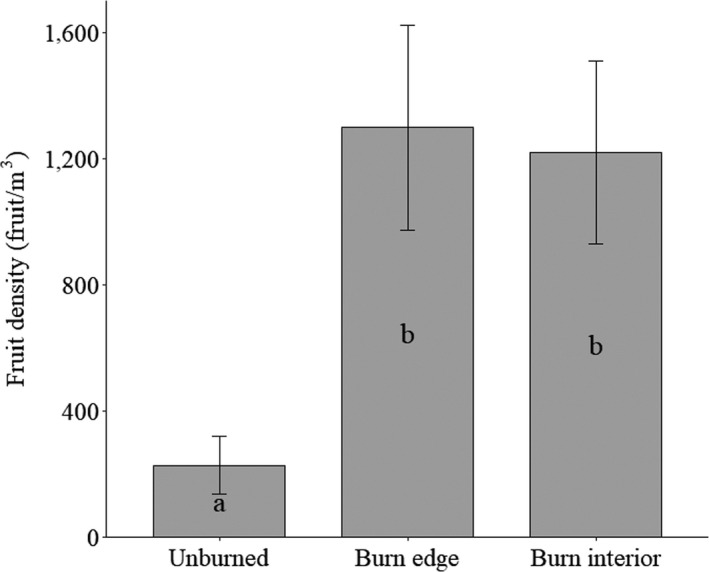
Average number of fruits per unit creosote shrub volume by burn condition and location ± *SE*. Significant differences (*p* < .05) are indicated with differences in letters

Mean fruit number differed between paired burn edge and unburned transects (Figure 5). Creosote on burn edge transects produced on average 4.7 times more fruit per shrub than shrubs on unburned transects (4,281 fruit vs. 919 fruit, *F* = 18, *p* = .0007; Figure 5). Mean fruit density was 5.7 times greater for shrubs in burn edge transects versus shrubs in adjacent unburned transects (1,297 fruit/m^3^ vs. 227 fruit/m^3^, *F* = 15.9, *p* = .03) (Figure 6). The effects of fire on fruit production were consistent (not statistically different) across the three years of data collection (Figure 5).

### 3.3 Growth rate and fruit number from burn edge and burn interior transects

Shrubs along burned edge transects and burned interior transects were not statistically different in stem diameter (*F* = 0.4, *p* = .6), number of stems (*F* = 3.9, *p* = .1), stem radial growth rates (*F* = 1.5, *p* = .3), height (*F* = 0.3, *p* = .6), vertical growth rates (*F* = 0.9, *p* = .4), fruit number (*F* = 0.1, *p* = .7), or fruit density (*F* = 0.06, *p* = .8).

## 4 DISCUSSION

Wildfires are increasing in size and frequency in North American deserts with varying effects on native plant density and composition (Abella, 2009; Brooks et al., 2004), but growth responses of resprouting plants after fire in North American desert shrublands are largely unstudied. Our study documents the positive effects of postfire environments on individual creosote stem growth and sexual reproduction. Our data support the conclusion that postfire environments increase creosote growth rates (Figure 4) and increase fruit number (Figure 5). However, distance to the fire perimeter did not affect growth rates or fruit number.

### 4.1 Stem age

The majority of sampled stems along burned transects dated postfire, while unburned stems began growing before the fire (Table 1). There were only two sampled stems from burned transects that dated from before the fire (16 and 13.6‐year old), indicating that only a small proportion of stems survived the fire. The majority of stems found in the burned areas of our study were determined to have begun growing after the fires and most likely as postfire resprouts (Bond & Midgley, 2001). Resprouting has been documented in creosote after fire (Abella, 2009) and varies depending on fire severity (Brooks, Minnich, & Matchett,^2018^; White, 1968). A study done in our same study area found that after the 2005 fires, around 21% of shrubs survived or resprouted along the burn edges, while around 3% survived or resprouted along burn interior transects (Lybbert et al., 2017). The fires in our study burned in June and July when mortality is highest and number of living sprouts has been shown to be the lowest (White, 1968). Along burn edges, burn severity ranged from low to moderate, while the burn interior transects had a higher proportion of moderate burn severity (Monitoring Trends in Burn Severity Program, 2017).

### 4.2 Growth rates in postfire desert communities

We found that creosote stems in burned areas grew faster than shrubs in unburned areas (Figure 4). Postfire resprouting shrubs have been shown to have rapid growth rates (Radosevich & Conard, 1980). Starch stored in the roots and root crowns of resprouting shrubs is vital for the production of new stem growth (Bowen & Pate, 1993; Neke, Owen‐Smith, & Witkowski, 2006). The creosote in burned areas had fewer stems; therefore, the increased growth rates we observed are likely in part due to the root system and nutrient reserves that previously provided for more stems (Bond & Midgley, 2001). The number and diameter of stem resprouts per plant depend on species (Neke et al., 2006), though some studies have found that for certain species, stem number decreases with increasing fire intensity and diameter increases with higher levels of stored nitrogen and nonstructural carbohydrates (Kabeya & Sakai, 2005; Moreno & Oechel, 1991; Neke et al., 2006). The fewer but larger stems we saw in burned areas could have been influenced by fire intensity and higher levels of postfire nutrients (Esque, Kaye, Eckert, Defalco, & Tracy, 2010). Also, despite the creosote in our burned study areas having fewer stems at their base compared to unburned areas, Horn et al. (2015) found that creosote in the same burned areas as our study had a higher canopy density (Leaf Area Index) than those in unburned areas. This emphasizes that the diameter of stems may alter canopy morphology between burned and unburned areas.

Environmental conditions can also affect the growth and survival of resprouting shrubs (Oechel & Hastings, 1983). Creosote has been documented to have higher growth rates with water addition treatments or combined water and nitrogen addition treatments in controlled studies (Sharifi et al., 1988). One possible effect of the fire is increased availability of water and nitrogen due to competitive release for soil resources since most of the neighboring shrubs were removed by fire (Horn et al., 2015; Valor et al., 2018). Our study sites had on average a 79% decrease in creosote on burned compared to unburned transect lines (Lybbert et al., 2017). This idea is further supported by studies that found that creosote shrubs grew larger after the neighboring shrubs were removed experimentally (Mahall et al., 2018) or increased growth rates for creosote with higher rainfall (Beatley, 1974; Gibson, Sharifi, & Rundel, 2004). Fire also creates a pulse of nutrients, especially under shrubs (Abella et al., 2009; Allen et al., 2011; Esque, Young, et al., 2010). This postfire increase in nutrients could also explain more rapid growth rates observed in our study (Fisher, Zak, Cunningham, & Whitford, 1988). Additionally, with lower shrub density after fire (Horn et al., 2015), it is possible that there may be a greater proportion of rodent burrowing underneath the regenerating shrubs, which increases soil nutrient levels, soil permeability, shrub size, and seedling survival that can increase soil resource availability linked to faster growth rates (Titus, Nowak, & Smith, 2002; Walker, Vrooman, & Thompson, 2015).

Increases in creosote growth rates after fire could have multiple effects on both the shrub itself and the surrounding environment. Similar to our study, Parmenter (2008) found that resprouting creosote reached their prefire heights 12 years after fire. It is unknown how long the growth rates of the regenerating shrubs in our study will continue. Larger creosote have been shown to be more prone to drought stress, but larger shrubs may be able to access deeper water sources (Franco, Soyza, Virginia, Reynolds, & Whitford, 1994). Also, the regenerating shrubs may be able to prevent some of the homogenization of nutrients across the landscape that is associated with disturbance and deterioration of fertile islands through loss of mature shrubs (Fuentes‐Ramirez et al., 2015; Klemmedson & Tiedemann, 1986). Fertile islands exist under desert shrubs and increase plant community diversity (Garcia‐Moya & McKell, 1970; Rostagno, Valle, & Videla, 1991; Schafer et al., 2012; Yeaton, 1978) although invasive *Bromus* grasses can also be facilitated by shrubs (Holzapfel & Mahall, 1999). Creosote in particular have been seen to have higher abundance of the invasive annual *Bromus rubens* L. on the north side of the shrub (Brooks, 2000), but creosote can also have negative impacts on other annual plants depending on precipitation and distance to canopy (Schafer et al., 2012).

### 4.3 Postfire reproductive response

Over the three‐year study period, fruit production was consistently higher along burned transects (Figure 5). Nitrogen additions have been shown to increase fruit production in creosote as well as other species (Breen & Richards, 2008; Fisher et al., 1988; Willson & Price, 1980). Conversely, water additions reduced the amount of fruit produced in creosote (Cunningham, Syvertsen, Reynolds, & Willson, 1979; Fisher et al., 1988). Increased fruit numbers could be also be driven by higher nitrogen availability following fire (Esque, Kaye, et al., 2010), competitive release for soil resources (Ehleringer, 1984; Horn et al., 2015), or higher levels of nutrients from root reserves or rodents (Kabeya & Sakai, 2005; Walker et al., 2015). Differences in canopy density and morphology could also contribute to the higher fruit numbers in burned areas (Figure 5; Horn et al., 2015).

An increase in fruit production per plant could partially compensate for fruit loss due to reduction in shrub density after the fire. However, total fruit per unit ground area is still lower in burned areas because of loss of shrub density (Lybbert et al., 2017), which may mean that fruits and seeds are not as evenly distributed across the landscape. Creosote seeds experience rodent predation (Boyd & Brum, 1983) and more seeds in a concentrated area could have impacts on seed predation and dispersal (Li & Zhang, 2007; Vander Wall, 2002). A more concentrated distribution of fruits and postfire plant community characteristics could also increase the dispersal of creosote seeds by wind (Maddox & Carlquist, 1985; Monty, Brown, & Johnston, 2013). However, studies showing that creosote takes many years to return to prefire densities could indicate that creosote establishment from seed is not always very effective after fire depending on environmental conditions or rodent predation (Abella, 2009; Engel & Abella, 2011; Steers & Allen, 2011).

### 4.4 Location within fire effect on growth rates (edges vs. interiors of large fires)

We found no statistical difference in growth rates on burned edges versus burned interior locations (Figure 4). In this region invasive ephemeral fuels, especially after high amounts of rainfall, provide enough fuel for fire to spread between shrubs as evidenced by the high rainfall preceding the 2005 fires (Brooks & Matchett, 2006). Fire severity and continuity, however, depend on the distribution and physical attributes of different invasive annual grasses present in the area (Brooks, 1999; Brooks & Matchett, 2006). It is possible that differences in fuel loads affected burn severity between the burn edge and interior, but there was not enough of a difference in burn severity to significantly change resprouting morphology or the growth rates (if burn severity affects growth rates). Since creosote growth is often water and nitrogen limited (Sharifi et al., 1988), the similar increased growth rates indicate that burn edges and the burn interior possibly had similar increases in water and/or nutrient additions to the shrubs whether through root reserves, total amount available, or through reduced competition.

## 5 CONCLUSION

In our study system, shrubs that resprouted after fire were able to do so vigorously. Fires have been shown to dramatically decrease the abundances of certain desert shrub species (Abella, 2009). This has led to concerns about increasing fire size and frequency in deserts due to shifts in invasive plant dominance. These fires could potentially lead to invasive grass‐fire cycles that result in a loss of ecosystem services and loss of biodiversity (Dantonio & Vitousek, 1992). However, our study indicates that fire can also provide opportunities for more rapid shrub growth and reproduction. The surviving shrubs, although fewer than prefire, could facilitate the re‐establishment of native plants after fire, stabilize the soil, and provide wildlife habitat (Bradley, Houghton, Mustard, & Hamburg, 2006; Esque, Schwalbe, Defalco, Duncan, & Hughes, 2003; Horn, McMillan, & St. Clair, 2012; Schafer et al., 2012; Soulard, Esque, Bedford, & Bond, 2013). While these increases in growth rates and reproduction occur after one fire, Brooks (2012) has shown that repeat fires further decrease abundance and diversity of native plants. The shrubs we studied were able to survive and resprout after one fire, but consecutive fires may limit re‐establishment success. If this happens any of the discussed benefits of these shrubs could be lost. More research is needed to know what the effect of repeat fires is on growth rates and reproduction of these shrubs.

## CONFLICT OF INTEREST

None declared.

## AUTHOR CONTRIBUTIONS

R. L. M. is co‐primary author and processed and analyzed samples, analyzed and interpreted the data, created figures, and wrote and edited the article. T. B. B. is also co‐primary author and helped in project design, collected data, analyzed the data, and edited the article. M. F. B. and S. G. K. analyzed samples, advised in data analysis, and edited the article. L. A. interpreted the data and edited the article. S. B. S. designed the project, collected data, interpreted the data, and wrote and edited the article.

## Data Availability

Dataset for individual shrubs and transects available at Dryad Digital Repository: https://doi.org/10.5061/dryad.t041bf0.
